# Innovate sodium alginate microneedle patches integrated with soft lidocaine invasomes: advanced strategies for oral ulcerative mucositis treatment via TNF-α/NF-κB pathways

**DOI:** 10.1007/s13346-025-01915-8

**Published:** 2025-07-17

**Authors:** Sammar Fathy Elhabal, Marwa Saeed Farahat, Mahmoud H. Teaima, Nahla A. Elzohairy, Mohamed El-Nabarawi

**Affiliations:** 1 Department of Pharmaceutics and Industrial Pharmacy, Faculty of Pharmacy, Modern University for Technology and Information (MTI), Mokattam, 11571 Cairo Egypt; 2 Department of Pharmaceutics and Industrial Pharmacy, Faculty of Pharmacy, Cairo University, Cairo, 11562 Egypt; 3 Department of Microbiology and Immunology, Faculty of Pharmacy, Modern University for Technology and Information (MTI), Mokattam, 11571 Cairo Egypt; 4 Air Force Specialized Hospital, Cairo, 19448 Egypt

**Keywords:** Oral ulcer, Mucositis, Invasomes, Microneedle patches, Sodium alginate, And Healing

## Abstract

Oral ulcerative mucositis (OUM) is a common painful disease that affects oral functions, such as eating or speaking leading to a low quality of life. This study aims to develop a novel strategy for relieving pain associated with OUM by using local anesthetics. Here, a hybrid dissolving microneedle patch integrated with lidocaine (Lido)-encapsulated invasomes (modified liposomes containing terpenes as penetration enhancers) depots are introduced for sustained Lido delivery, reduced dosing frequency, and improved patient compliance. Different Lido-loaded invasomes formulations were developed using design expert^®^ software to study the effects of different type terpenes (Limonene, Cineole, Camphor) and their concentration using a thin-film hydration approach. Dissolving microneedle (MN) patches made of sodium alginate (SA), Glycerol and polyvinyl alcohol (PVA) via the casting method. Optimized invasomes formulations containing cineole exhibited excellent stability, a high entrapment efficiency of 83.5%, and a nanoscale size of approximately 295 nm. The incorporation of SA/PVA with 1% glycerin MNs resulted in effective mucosal penetration, rapid dissolution within 10 min, and significant mechanical strength. Research conducted in-vitro and ex-vivo demonstrated enhanced permeation and a significant increase in lidocaine release, achieving 95% within 24 h. In-vivo evaluations demonstrated substantial pain relief, reduced inflammation (evidenced by decreased TNF-α and NF-κB levels), enhanced anti-inflammatory IL-10 expression, and modulation of angiogenesis via VEGF downregulation, leading to accelerated mouth healing with complete epithelial restoration. This hybrid system significantly improves drug delivery and patient comfort by aiding in biocompatibility, Mucoadhesion, and healing. This innovative system transcends traditional anesthetic administration, providing a painless and targeted therapeutic platform that improves OUM management.

**Graphical Abstract:**

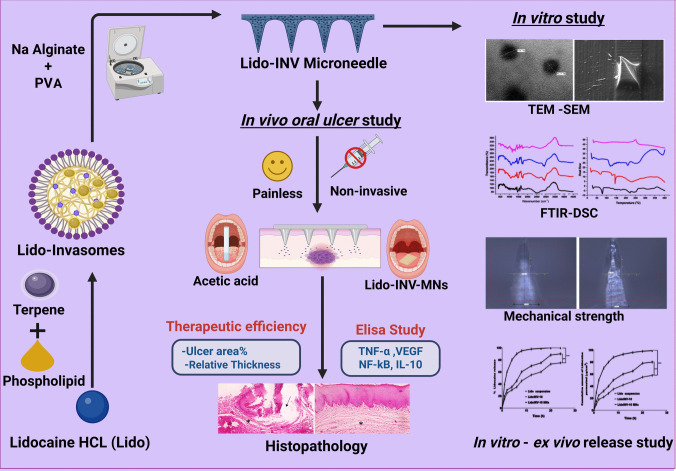

## Introduction

Oral ulcerative mucositis (OUM) is one of the most common complications experienced by cancer patients undergoing chemotherapy, radiotherapy, or a combination of the two. The OUM is defined as an alteration of the oral mucosa that results in inflammation and ulcerative lesions[1, 2]. Mucositis symptoms can be mild or so bad that they need to be treated in the hospital. Some of the signs are swelling, pain, ulcers, bleeding, a dry mouth, burning, and trouble speaking and swallowing[3]. Because chewing, swallowing, taste, and breath are compromised, mucosal ulcers and pain can make eating and drinking difficult[4, 5]. A painful disorder, oral ulcerative mucositis can interfere with speech and eating among other oral activities. Severe OUM-induced pain is treated by rinsing, coating, or patching anesthetics and antibiotics. Giving drugs to the OUM area could cause a short duration of action, diminished tactile sensation, inadequate analgesia, and pain induction. Lidocaine induced hypoalgesia in healthy mucosa with a temporary analgesic effect[6, 7]. In contrast, prior animal studies indicated that topical indomethacin reduced OUM-induced pain but did not influence mechanical allodynia. Linalool presents a potential option for pain management, as its aroma may alleviate discomfort associated with opioid use without direct contact with the affected region[8]. The treatment objectives include pain reduction, acceleration of healing, and prevention of infection onset. Individuals utilize"magic mouthwash,"viscous lidocaine, and benzocaine to alleviate discomfort associated with speaking and eating. Systemic analgesics such as lidocaine, acetaminophen, ibuprofen, or opioids can effectively manage severe pain[9]. Honey and the keratinocyte growth factor palifermin facilitate mucosal healing. Consuming uncomplicated foods and maintaining hydration aids in preventing irritation while ensuring nutritional adequacy. Avoiding spicy foods, alcohol, tobacco, and oral cryotherapy during chemotherapy, along with maintaining good oral hygiene using a soft toothbrush and alcohol-free mouthwash, are essential preventive measures[10].

Lidocaine HCl is a Skin-BCS (skin-related biopharmaceutics classification system) Class III drug as a lipophilic compound, has high solubility (0.054 g/mL) with poor permeability (LogP = 2.3) through the skin, which is the main barrier to its use in transdermal administration[11]. Lidocaine is a local anaesthetic that is commonly used in clinical practice to reduce or eliminate pain, both during surgery and for the treatment of other types of acute or chronic pain like dentistry, and veterinary care acting by blocking sodium channels in nerve cells stop pain signals[12]. Lidocaine is used in emergency medicine to relieve severe pain and stabilize irregular heart rhythms, particularly ventricular arrhythmias. This drug is typically given as an injection through the intramuscular, intravenous, or subcutaneous routes, which may increase bioavailability. However, the injection route has some drawbacks, such as pain when administered, the risk of infection, local irritation, trypanophobia (an excessive fear of needles), the need for medical personnel who are experienced in drug administration, problems with medical waste, and high storage costs[13]. Topical lidocaine creams and gels numb the skin, reducing pain and itching from minor cuts, scrapes, and burns. Sprays and gels numb mucosal surfaces during medical procedures. Lidocaine mouth rinses reduce oral ulcerative mucositis pain, making eating and speaking easier. Lidocaine and other painkillers are often used together to improve patient comfort and experience during Botox, laser, and dermal filler treatments[14].

Nanotechnology has led to the creation of nanomaterials that can carry active substances and make them work better. In fact, nanoscale engineering can get around the problems with current drug delivery methods. A good drug delivery system (DDS) acts as a protective shield for the payload, preventing premature decomposition in the biological environment while increasing bioavailability, blood circulation duration, cellular absorption, and therapeutic activity over time[15]. Recently developed liposomes, which are novel vesicular systems containing lidocaine. They were discovered to be capable of self-association, resulting in stable nanosized vesicle structures.

Invasomes (INV) are novel class of liposomes that contain terpenes as natural penetration enhancers (PE), the ingredients include phosphatidylcholine, ethanol, and a standard terpene blend of cineole, citral, and d-limonene in a 45:45:10 ratio[16]. The new vesicular carrier system improves temoporfin permeation across the stratum corneum compared to traditional liposomes[17]. Many medications, including curcumin, isradipine, nimsulide, and avanafil, have been shown to improve skin penetration and deposition when invasomes are present[18, 19]. In comparison to conventional liposomes, invasomes perform better due to the presence of terpenes and ethanol. Because of the interactions between the stratum corneum and its lipids, the stratum corneum becomes fluidized and loses its taut structure. On top of that, it makes vesicles more pliable, so they can pass through damaged SC[20].By creating new bonds between terpenes and ceramides and replacing hydrogen bonds between ceramides in SC, terpenes improve penetration. Comparing this to traditional liposomes[21].discovered that the vesicle integrity is weakened, ethanol, terpenes, and vesicular carriers work in concert to improve drug penetration through invasomes.

Microneedles (MNs), which are also known as microneedle array patches (MAPs), are a promising alternative to transdermal patches for getting more drugs into the skin. A substrate holds these very small needles in place[22]. The needles are 50–900 μm tall and have a density of up to 2000 MN/cm^2^.They make microconduits by puncturing the stratum corneum when they go into the dermis. MNs are classified into several types: solid, hollow, coated, dissolving, and hydrogel-forming. Dissolving MNs (DMNs), which are made from biodegradable polymers containing targeted drugs, dissolve when exposed to interstitial fluids after insertion, allowing for drug release, because of the water-soluble properties of the polymer matrix, hydrophilic molecules frequently associate with MNs[23]. Hydrophobic compounds are difficult to incorporate into MNs because of their low solubility in water. To overcome this challenge, nano-sized particles must be created, which increases solubility and promotes uniform drug distribution within the DMAP matrix.MN patches are made up of tiny needles that are affixed to the patch. MN is a useful application because it can pass through the stratum corneum painlessly[24].

Both biodegradable and non-biodegradable materials are available for (MN) fabrication. Materials such as amylopectin, polyvinyl pyrrolidone, chitosan, eudragit, hyaluronic acid, polyvinyl pyrrolidone, polylactic acid, and PLGA are used to make biodegradable MNs.Because accidental breakage during insertion can result in complications like sepsis, biodegradable MNs are preferred over non-biodegradable ones[25].

Various polysaccharides including pectin, chitosan, konjac, carrageenan, and some plant polysaccharides allow one to offset the lack of sodium alginate by forming microcapsules[26]. Natural polysaccharide polymer sodium alginate (SA) is biocompatible, non-toxic, biodegradable. Among the several advantages of polymers are gelling, swelling, and adhesive qualities.

Sodium alginate, a natural polysaccharide derived from brown seaweed, is biocompatible, nontoxic, and biodegradable. It comprises two monosaccharide units: mannuronic acid (M) and guluronic acid (G), which are connected by 1,4-glycosidic bonds. The mannuronic to guluronic acid ratio (M/G ratio) influences sodium alginate's gelation properties and behaviour in drug delivery systems. The molecular weight of sodium alginate typically ranges between 20,000 and 50,000 Da. When SA interacts with cationic ions, particularly calcium ions, it cross-links and forms a stable gel structure. Furthermore, the degree of substitution and branching of the alginate can influence its properties in drug delivery applications, such as viscosity and the rate of drug release. SA ability to produce gel formation in contact with cationic ions is often utilized in enteric drug formulation technology[27]. SA modified during the cross-linking process results in formulations that are insensitive to gastric acid and pass unchanged into the intestinal environment. In the intestinal environment, the higher concentration of phosphate ions causes the SA matrix to relax, ensuring intestinal drug release[28].

Chitosan (Ch) is a great carbohydrate polymer, a natural biopolymer for treating ulcers because it kills germs and helps wounds heal[29]. Biocompatibility and biodegradability make chitosan useful for ulcer healing. It activates platelets to initiate hemostasis and ulcer stabilization [30]. Chitosan increases fibroblast migration and proliferation, promoting tissue regeneration. Collagen deposition strengthens wound closures. Chitosan's moisture retention prevents wound drying and stimulates tissue repair[31].

Building upon this background, the present study aims to develop a strategy to minimize pain in the use of local anesthetic agents. The development and optimization of lidocaine-loaded dolinvasomal formulations incorporated into dissolving sodium alginate/polyvinyl Alcohol (SA/PVA) microneedles for the enhanced treatment of oral ulcerative mucositis. The optimized the invasomal formulations using Box-Behnken design, ensuring robust statistical analysis of critical quality attributes (particle size, polydispersity index, zeta potential, and entrapment efficiency). The fabrication of microneedles was carefully carried out, and their mechanical strength, penetration ability, drug content, dissolution behavior, and structural integrity were thoroughly characterized using a variety of standard techniques (SEM, DSC, FTIR).

Therefore, this study conducts in vitro, ex vivo, and in vivo evaluations for oral ulcer-healing with detailed biochemical and histopathological evaluations potential of the proposed Lid o-INV 10 MNs, providing a foundation for future clinical applications.

## Materials and methods

### Materials

El-Nile Company for Pharmaceuticals in Cairo, Egypt generously provided lidocaine HCl. Lipoid GmbH of Germany provided soyabean phosphatidylcholine (Phospholipon^®^ 90 g). Sigma-Aldrich Chemical Co., Germany supplied PVA (Polyvinyl alcohol, (C₂H₄O)ₙ, Mn ≈ 85,000–146,000), sodium alginate Sodium alginate (CAS. 9005–38-3, viscosity: 200 ± 20 mPa.s, M/G ratio of 1, molecular weight: 8.65 × 10^4^ Da, purity: 99.7%), Camphor, methanol, and ethanol (HPLC grade). Limonene, cineole, acetic acid, and Co were obtained from Alfa Aesar in Munich, Germany. El-Gomhouria Pharmaceutical Co., Cairo, Egypt, supplied Parafilm M^®^ (Bemis Company Inc., Neenah, WI, USA), methanol, chloroform (HPLC grade), Tween 80, sodium dihydrogen phosphate, and disodium hydrogen phosphate. Millipore, MA provides deionized water through its MilliQ^®^ purification system.

## High-performance liquid chromatography (HPLC) analysis of lidocaine

Lidocaine was analyzed using reversed-phase HPLC on a Water Agilent 1260 series perform HPLC analysis. The separation was performed with a Shiseido column (4.6 mm × 250 mm i.d., 5 μm). The mobile phase was made up of 835 ml of water and 65 ml of NaOH 1 M with a pH of 2, which was then combined with phosphoric acid to reach a total volume of 1L. The flow rate was 1 ml/min. The mobile phase was programmed to be isocratic. The DAD detector was observed at a wavelength of 208 nm. Each of the sample solutions had an injection volume of 20 μl and column temperature was maintained at 30 °C[32, 33].

## Preparation of lidocaine-loaded invasomes and soft nanovesicles

**Fig. 1:**
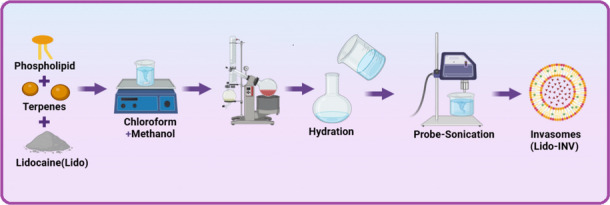
Graphical thin-film hydration method for the fabrication of lidocaine-loaded invasomes (Lido-INV)

Lidocaine invasomes (Lido-INV) preparations were made using the conventional thin film method. Lidocaine (1 mg/mL), phospholipid 90G (10% w/v), and terpenes (limonene or cineole) were added to a dry clean rotary evaporator flask and dissolved in a 2:1 (v/v) chloroform–methanol solution. A rotary evaporator let the organic solvent evaporate, 500 mbar was the vacuum used for five minutes; then, for five minutes, 200 mbar; last, for one hour, 40 mbar, one overnight using a vacuum, organic solvents are eliminated. The thin lipid film was then hydrated with a 10% v/v ethanolic phosphate buffer solution (pH 7.4) by rotating at 100 rpm for 1 h at 60 °C[34, 35]. Probe sonication was used at 4 °C (with an ice cube bath added to the suspension) at 60% frequency for 5 min to reduce particle size and homogenize completely as shown in Fig. 1.

## Formulation optimization

Using the box-Behnken design, the effect of terpene types and terpene conc (X1 and X2 respectively) on the dependent variables, namely, particle size (PS), polydispersity index (PDI), zeta potential (ZP), and entrapment efficiency (Y4), was statistically evaluated. Table 1 shows three levels for each factor. ANOVA was used to analyze all data, with a significance level of (p < 0.05). Using Box-Behnken, the statistical analysis was followed by a calculation of the desirability to determine the best formula for future research study.

**Table 1:** Box-Behnken design for optimization of lidocaine-invasomes

Factors (independent variables)	Levels		
X_1_: Type of terpene	Limonene	Cineole	Camphor
X2: Concentration of terpene	0.5%	1%	1.5%
Responses (Dependent variables)	**Constraints**		
Y_1_: PS (nm)	Maximize (absolute value)		
Y_2_: PDI	Minimize		
Y_3_: ZP	Minimize		
Y_4_: EE (%)	Maximize		

**Abbreviations**: EE%; entrapment efficiency percent, PS; particle size, PDI; polydispersity index, and ZP; zeta potential

## In vitro characterization of lid-loaded invasomes

### Particle size, zeta potential, and polydispersity index determination

The Malvern Zetasizer (HAS 3000; Malvern Instruments, Malvern, UK) was used to measure the average size of invasome particles, polydispersity index, and zeta potential using dynamic light scattering. The specimen was placed in a quartz cuvette and diluted with distilled water. Size measurements were taken at 25 ± 1 °C with a scattering angle of 90°. For each formulation, observations were recorded three times. The particle shape was viewed through a Philips CM10 transmission electron microscope (Philips Research, Hamburg, Germany). The samples were dried on a carbon-coated grid before being stained with an aqueous solution of phosphotungstic acid. Following drying, the specimen was examined under a microscope at 10–100 k-fold enlargements, using a 100 kV accelerating voltage[36].

### Entrapment efficiency determination

To separate free, unentrapped drugs from Lido-loaded nanovesicles, they were centrifuged at 12,000 rpm for 1 h at 4 °C in a cooling centrifuge. The concentration of Lido in the clear supernatant was determined using HPLC as previously stated. The total Lido content of each formulation was determined after vesicle lysis with absolute methanol, sonification for 10 min, followed by HPLC analysis[37]. Entrapment efficiency was calculated indirectly using the Eq. (1):

1 $$\mathrm{EE}\%=\frac{Total\;drug\;content-Free\;unentrapped\;drug}{Total\;drug\;content}\times100\%$$

## Statistical analysis

The Box-Behnken statistical design was implemented with Design-Expert software (version 7, Stat-Ease Inc., USA). Table 2 summarizes the design and displays two factors, X1 (Span 60: EA ratio (w/w)) and X2 (EA Type), each with three levels. The best formulation was selected after reviewing experimental data and performing a desirability calculation.

**Table 2:** Experimental runs, independent variables, and measured response of the Box-Behnken experimental designs of lidocaine-invasomes

	Factors			**Responses**			
Run	X1: Concentration of penetration enhancer	X2: Phospholipid concentration %	X3: Type of penetration enhancer	**Y1:** PS (nm)	Y2: PDI (nm)	Y3: ZP (mV)	Y4: EE (%)
INV1	0.5	1	Limonene	410 ± 0.03	0.441 ± 0.03	−28.78 ± 0.05	66.32 ± 0.09
INV2	0.5	1	Camphor	394.8 ± 0.21	0.378 ± 0.01	−31.43 ± 0.23	63.74 ± 0.54
INV3	1	1	Cineole	388.2 ± 0.33	0.311 ± 0.02	−22.17 ± 0.51	76.3 ± 0.37
INV4	1	5	Cineole	552 ± 0.08	0.542 ± 0.11	−27.32 ± 0.43	69.32 ± 0.98
INV5	1.5	5	Camphor	560.1 ± 0.32	0.478 ± 0.09	−24.61 ± 0.09	54.32 ± 0.47
INV6	0.5	5	Camphor	279.3 ± 0.41	0.381 ± 0.07	−24.54 ± 0.43	72.43 ± 0.62
INV7	1.5	1	Limonene	338.6 ± 0.21	0.266 ± 0.04	−30.17 ± 0.41	70.87 ± 0.51
INV8	1.5	3	Cineole	434.1 ± 0.11	0.425 ± 0.02	−30.31 ± 0.22	77.63 ± 0.91
INV9	0.5	5	Limonene	510.3 ± 0.25	0.491 ± 0.01	−23.15 ± 0.41	64.12 ± 0.83
**INV10**	**0.5**	**3**	**Cineole**	**295.8 ± 0.61**	**0.267 ± 0.01**	**−30.11 ± 0.98**	**83.57 ± 0.43**
INV11	1.5	1	Camphor	489.8 ± 0.61	0.405 ± 0.03	−31.16 ± 0.52	61.87 ± 0.81
INV12	1	3	Limonene	478 ± 0.09	0.498 ± 0.06	−31.15 ± 0.41	60.98 ± 0.45

**Abbreviations**: EE%; entrapment efficiency percent; PS; particle size, PDI; polydispersity index, SD: standard deviation, ZP; zeta potential, and bold indicated optimized formula

## Characterization of optimum lido-loaded invasomes formulation

### Transmission electron microscopy (TEM)

The morphological evaluation of the systems was designed to determine structural properties such as lamellarity, size, shape uniformity, and the presence of aggregated Lido-loaded INV. A single drop of the properly diluted dispersion was placed on a 300-mesh carbon-coated copper grid and allowed to settle for 3 to 5 min. The excess fluid was removed with filter paper, and the grid was allowed to air dry at room temperature for 10 min before being examined under an 80 kV transmission electron microscope (Jeol JEM 1230; Tokyo, Japan)[38, 39].

### Fourier transform infrared spectroscopy (FT-IR) analysis

The FT-IR spectral analysis aimed to confirm the likely physicochemical compatibility and chemical intermolecular interactions between Lido, invasomes without Lido, and Lido-INV. Spectral scanning in the wavelength range of 4000 to 400 cm-1 was performed with an FTIR spectrophotometer (Shimadzu; Kyoto, Japan)[40].

### Differential scanning calorimetry (DSC)

The thermal properties, crystallinity, and interactions between Lido invasomes without Lido and Lido-INV were investigated using differential scanning calorimetry (DSC, Shimadzu Corporation, Kyoto, Japan). A sample of Lido, invasomes without Lido, and optimal Lido-loaded invasomes formulation was analyzed, and differential scanning calorimetry (DSC) thermograms were recorded at temperatures ranging from 25 to 250 °C, using nitrogen as the purging gas at a flow rate of 100 ml/min[41].

## Stability study

The stability of the optimal Lido-loaded invasomes formulation was evaluated by observing changes in PS, PDI, ZP, and EE%. For three months, the optimal Lido-loaded invasomes formulation was stored at 4 and 40 degrees Celsius. As previously stated, we assessed PS, PDI, ZP, and EE% after three months using three independent measurements[35, 42].

## Freeze-drying of lidocaine-loaded Invasomes solutions

The INV dispersions were loaded with Lido, frozen, and lyophilized in the Novalyphe-NL 500 lyophilizer for 24 h at –45 °C and 7 m bar pressure. Solid-state studies were then carried out on the resulting lyophilized INV, as well as their components and the physical mixture[23].

## Design and fabrication of sodium alginate, polyvinyl alcohol (SA/PVA)-MNs loading freeze-dried Lido-loaded INV10

**Fig. 2:**
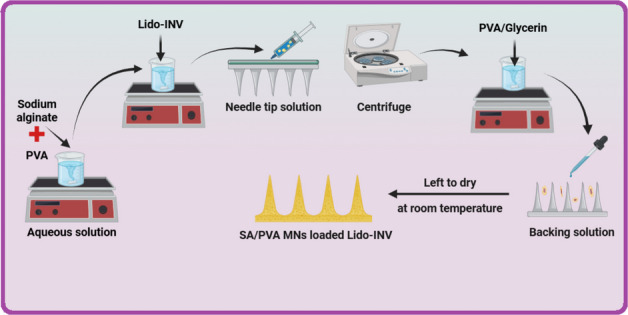
Preparation method of sodium alginate/polyvinyl alcohol microneedles loaded with lidocaine-loaded invasomes (SA/PVA-MNs loading Lido-INV)

MNs were cast using a polydimethylsiloxane (PDMS) mold (5 × 5 arrays, base diameter 100 μm, depth 300 μm). As shown in Fig. 2, an aqueous solution containing various ratios of lyophilized Lido-INV, sodium alginate, polyvinyl alcohol (PVA), and 1% w/w glycerin was cast into the MN molds, according to Table 3. After removing excess formulation, the MNs were centrifuged at 6000 × g for 5 min and dried at room temperature (RT) for 24 h. A base plate was created by pouring a drug-free polymeric solution containing varying ratios of sodium alginate, polyvinyl alcohol (PVA), and 1% w/w glycerin into MN molds. The MNs were centrifuged at 6000 × g for 5 min and allowed to dry at room temperature for 24 h.

**Table 3:** Fabrication of SA/PVA-MNs loading freeze-dried lidocaine-invasomes

Formulation	Lido-INV 10 (% w/w)	Sodium Alginate (% w/w)	PVA (% w/w)
MNs1	40%	20%	12%
MNs2	30%	25%	15%
MNs3	50%	15%	10%

Lido-INV: lidocaine loaded invasomes, SA: Sodium Alginate, PVA: Polyvinyl Alcohol, and MNs: Microneedle-all formulation containing 1% w/w glycerin

## Physical characteristics of the microneedles

### Mechanical strength and penetration capability test

This evaluation looked at the mechanical strength and penetration efficacy of microneedles. The tests involved placing the various MNs on eight layers of Parafilm M^®^, a pliable thermoplastic sheet made of an olefin-type material that mimics the thickness and texture of human skin. MNs were subjected to pressure equal to 32 N (3.2 kg), or average adult thumb pressure, for 30 s. The shape and height of the MNs were measured, as well as the number of holes in each layer of Parafilm M. The equations below were used to calculate mechanical strength and penetration capability[43, 44].

2 $$\%\mathrm{Height}\;\mathrm{Reduction}=\frac{Initial\;height-Final\;heigh}{Initial\;height}\times100\%$$

3 $$\%\mathrm{Penetration}\;\mathrm{capacity}=\frac{\mathrm{Number}\;\mathrm{of}\;\mathrm{holes}}{\mathrm{Total}\;\mathrm{needles}}\times100\%$$

### Studies on drug content

An array of Lido-INV-loaded SA/PVA-MNs magnetic agitators was dissolved for one hour in distilled water containing 2.5% Tween 80 while being stirred at 300 revolutions per minute. The resulting solution was diluted with methanol and sonicated for five minutes to fully dissolve the INV from the fabricated Lido-INV-loaded SA/PVA-MNs. The drug content of the produced MNs was determined using HPLC analysis, as previously mentioned[23, 45].

### Dissolution time test

The dissolution time of MNs was determined using a full-thickness chicken pouch membrane model. Manual thumb pressure was used to apply MNs to the chicken pouch membrane. A 5 g load was used to ensure a long-lasting attachment. MNs detached from the skin at 2, 4, 6, and 8-min intervals, allowing for a dissolution time examination[46].

### Water loss on drying (LOD)

To determine the limit of detection (LOD), 0.5 g of MNs polymers was accurately weighed into a mold. The polymers were dried in a desiccator and weighed again after 48 h[47]. The data from these measurements were analyzed using the following equations:

4 $$\%\mathrm{LOD}=\frac{Initial\;weight-Final\;weight}{Initial\;weight}\times100$$

## Characterization of optimized microneedle

### Scanning electron microscopy (SEM)

The morphology of the Lido-INV-loaded SA/PVA-MNs patch was investigated using a scanning electron microscope (SU8010, HITACHI, Japan). The Lido patch was affixed to a microscope carrier, and visualization was conducted at 6 kV with a magnification of 150x.

### Differential scanning calorimetry (DSC)

The thermal properties, crystallinity, and interactions among Lido-INV-loaded SA/PVA-MNs patch were examined using differential scanning calorimetry (DSC, Shimadzu Corporation, Kyoto, Japan), at temperatures ranging from 25 to 250 °C, utilizing nitrogen as the purging gas at a flow rate of 100 ml/min as mentioned before.

### Fourier transform infrared (FTIR) analysis

An FTIR spectrometer was used to record the room temperature FTIR spectra of a Lido-INV-loaded SA/PVA-MNs patch and its constituent parts between 500 and 4000 cm^−1^ (Tensor II, Germany, Bruker).

## Drug release studies

### In vitro drug release study

The in vitro release of Lidocaine from Lido-solution, Lido-INS 10, and Lido-INV-loaded SA/PVA-MNs was measured with a USP dissolution testing apparatus type II (Hanson Research Corp., CA, USA). To ensure sink conditions, the dissolution media consisted of 100 mL of pH 6.8 phosphate buffer with 25% methanol. The formulations (10 mg of Lidocaine) were placed in two open-ended tubes: one sealed with a cellulose membrane and the other attached to the shaft of the dissolution device. The dissolution media were kept at 37 ± 1 °C, and the shaft rotated at 50 rpm. At different time intervals (0.25, 0.5, 1, 2, 4, 6, 8, 10, 12, 24 h), 1 mL samples from the dissolution media were taken and replaced with an equal volume of fresh medium. Lidocaine concentration was determined using the previously mentioned HPLC technique at a wavelength of 208 nm. The steady-state flux (Jss) and percent drug release after 24 h (Q24%) were calculated. The in vitro release data were kinetically evaluated using four different release models (Higuchi, first-order, Peppas, and zero-order). Release studies were performed in triplicate (n = 3)[26].

### Ex vivo permeation study

Ex vivo permeation studies were conducted using a modified Franz diffusion cell. The chicken pouch membrane was attached to the donor and receptor compartments and modeled after human buccal mucosa. Ten mg of each Lido-soluble lido-INV-loaded SA/PVA-MN (equivalent to ten mg of drug) was precisely weighed and placed in the donor compartment. The receptor compartment was filled with 25 millilitres of a pH 6.8 phosphate buffer solution containing 25% methanol. This would help to achieve sink condition. The sample was kept spinning magnetically at 100 rpm and 37 ± 1 ◦C. Every hour for 24 h, one millilitre of permeation media was removed from the receiving cell and replaced with an equal volume of fresh media[21, 48]. There were three replicate experiments. After filtering through a 0.45 µm membrane, the samples underwent validation HPLC analysis. Time was plotted against the lidocaine concentration that passed through the Fresh Chicken Pouches membrane. The apparent vaginal permeable coefficient (cm/h) was calculated using the equation Kp = Jss/C0, where C0 was the initial drug concentration (µg/h.cm^2^) and Jss (steady stat flux) was the slope of the linear part (µg/cm^2^). After carefully washing the Fresh chicken pouches'membrane three times with deionized water, 20 mL of methanol was used to soak it overnight and then sonicated for 15 min in a sonicator bath at the end of the ex vivo permeation experiment. Samples were collected, passed through a 0.45 µm membrane, and analyzed using a validated HPLC technique to determine the amount of lidocaine deposited in the mucus tissue after 24 h[49].

## In vivo studies

### Animals

All of the principal investigators (PI) for animal studies were given approval by the Research Ethics Committee of the Faculty of Pharmacy at Cairo University. Every single experiment that involves animals is conducted in accordance with the ARRIVE standards as well as the instructions provided by the National Institutes of Health (NIH Publications No.8023, revised 1978) regarding the care and use of laboratory animals. A total of thirty mature male New Zealand rabbits, weighing between 2.5 and 3.0 kg, were obtained, were obtained from the animal center of the Faculty of Pharmacy at Cairo University. These rabbits were randomly assigned to five groups, for a total of six groups. In addition, they were accustomed to having three on each cage and were maintained in a 12:12 light cycle with a temperature of 24 ± 1 °C and a humidity of 50–55%. Following the completion of the experiments, the animals were sedated with ketamine (50 mg/kg, i.m.) and euthanized by intravenous injection of sodium pentobarbital (150 mg/kg), in accordance with IACUC ethical guidelines.

### Induction of oral ulcerative mucositis and experimental design

Thirty mature male New Zealand rabbits were randomly assigned to five groups (n = 6), weighing 2.5–3.0 kg, and were utilized to establish an oral ulcer model on the tongue. Before the formation of the ulcers, the rabbits administered anesthesia (intramuscular; ketamine 35 mg/kg, xylazine 5 mg/kg). A circular filter paper (diameter: 6 mm) was saturated with 15 µL of 50% acetic acid. The acid-saturated paper was applied to the rabbit's labial tongue tissue for 60 s to induce circular ulcers. These groups included: Group I (normal group, negative control), Group II (disease model group, positive control), and three treated groups: Group III (treated with a lidocaine solution), Group IV (treated with lidocaine loaded invasomes), and Group V (treated with an optimized microneedle patch), all treated groups with received 1% of Lido with different formulation. All treatments began 24 h after ulcer induction, with formulations applied topically to the ulcer surface twice daily for 5 days. Mucosal ulcer healing in the rabbits was observed and recorded daily for five days after modeling, and images were taken to document the changes. ImageJ was used to measure the ulcer area and track oral ulcer healing on days one through five[26].

### Blood sampling and biochemical assessment of inflammatory biomarkers

After the experiment was completed, the rabbits were given anesthesia, blood samples were taken, and then they were then decapitated to put an end to their suffering. After drawing a portion of the blood, it was placed in plain tubes so that the serum could be separated and examined separately. The levels of Vascular Endothelial Growth Factor (VEGF), Nuclear Factor kappa-light-chain-enhancer of activated B cells (NF-κB), Interleukin-10 (IL-10), and Tumor Necrosis Factor-alpha (TNF-α) were measured through the utilization of ELISA tests. Using commercial ELISA kits, the levels of VEGF (EK720346, AFG Bioscience^®^, USA), NF-κB (ab285316, Abcam, USA), IL-10 (ab100765, Abcam, USA), and TNF-α (ab236712, Abcam, USA) were determined. The measurements were carried out in accordance with the instructions provided by the manufacturer. At a wavelength of 450 nm, absorbances were measured by employing the testing procedures described in the manuals. For the relevant standard curve (supplementary data), various sample concentrations were computed after being taken into consideration. All the samples were stored at −80 degrees Celsius until they were used[50].

## Histopathological examination

The rabbits that had been treated were put under anesthesia and then sacrificed on day five. Following the fixation of the oral mucosal ulcer tissue with 10% paraformaldehyde for a period of twenty-four hours, the tissue was then dehydrated in graded ethanol, embedded in paraffin compound, and sectioned. Hematoxylin and eosin (H&E) staining was performed on these sections to evaluate the healing process and the amount of collagen that was deposited in the ulcerated region[26, 51].

## Results and discussion

### Invasomes characterization

#### Particle size, PDI, zeta potential, and encapsulation efficiency

##### Effect on Particle Size (PS)

**Fig. 3:**
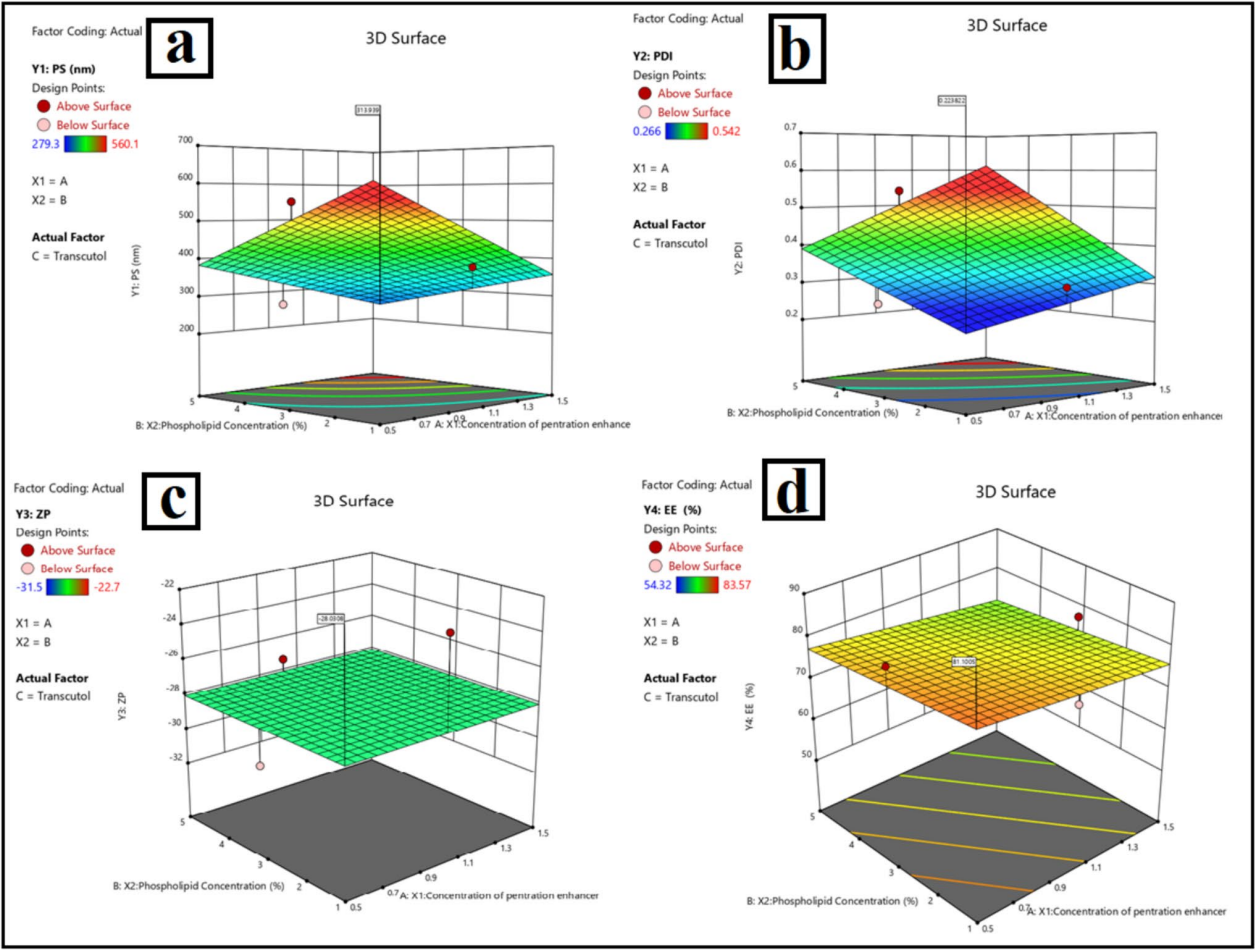
Three-dimensional response surface plot for the effect of type of penetration enhancer, the concentration of penetration enhancer, and phospholipid concentration on invasomes. **Note**: **a**: particle size, **b**: polydispersity index, **c**: zeta-potential, and **d**: entrapment efficiency

As shown in Table 2, and Fig. 3 formulations F1 to F12 were successfully prepared for Lido-loaded invasomes. Comparing the effect of the three different penetration enhancers terpenes limonene, cineole and Camphor on the particle size of soft nanovesicles revealed almost identical behaviors of both cineole and cineole exhibiting comparable effects on the particle size of the formulations, with only slight differences across the concentration range of 0.5% to 1.5%. Cineole produced the smallest vesicle sizes at low concentrations (e.g., 388.2 nm in INV3, 295.8 nm in INV10) when compared to limonene and camphor. The moderate log P (~ 2.8) allows for efficient distribution between the hydrophobic bilayer and aqueous environment, resulting in tight vesicle packing. With the highest *P* (~ 4.5), limonene is highly lipophilic and preferentially insert into the hydrophobic bilayer, resulting in membrane fluidization and expansion and hence larger vesicles (e.g., INV1: 410 nm, INV12: 478 nm). With a lower log *P* (~ 2.4), camphor somewhat disturbs the bilayer and produces intermediate effects on particle size. As terpene concentration increased, PS also increased across all types. The higher the terpene content, the more disruption occurs within the phospholipid bilayer, causing swelling, vesicle fusion, or aggregation, regardless of log *P*. However, this effect was more pronounced with limonene, consistent with its strong lipophilicity and bilayer-disordering potential. Similar findings were obtained by Saffari et al. (2016), whereby the effect of Cineole-containing vesicles on the lipid bilayer structure caused reduced particle sizes[52]. These findings show that Cineole is useful in lowering vesicle size relative to terpene-based formulations, so underscoring its function in improving vesicle stability while producing smaller, more homogeneous particles.

### Effect on polydispersity index (PDI)

Lower PDI values (0.267–0.311) consistently found in cineole-containing invasomes suggested more homogeneous vesicle populations. The moderate log *P* of cineole enables balanced insertion into the bilayer without too disruptive effect. Higher PDIs (e.g., INV1: 0.441, INV5: 0.478) clearly show that limonene (high log P) and camphor (rigid cyclic structure) generate less uniform vesicles. Particularly with highly lipophilic terpenes like limonene, which cause unequal bilayer fluidization and vesicle destabilization, vesicle heterogeneity raised with concentration (higher PDI)[19].

### Effect on zeta potential (ZP)

Zeta potential (ZP), is an important factor that influences the stability of vesicles. The newly obtained findings indicate that all formulations exhibited negative ZP values, ranging from −22.17 mV to −31.43 mV, which indicates that the colloidal stability of the formulations was moderate. INV2 (−31.43 mV, Limonene) and INV11 (−31.16 mV, Limonene) exhibited the lowest values. Camphor and limonene invasomes had lower zeta potential values (− 31 mV) than cineole (− 22 to − 30 mV). The higher surface charge observed with limonene and camphor can be attributed to their interaction with the phospholipid headgroups on the vesicle surface. High log *P* limonene causes it to preferentially embed in the bilayer core, so possibly exposing more negative phospholipid headgroups and raising surface charge. Though less lipopholic, camphor may align close to the bilayer interface to boost surface charge. Not greatly changing surface charge, Cineole with a more balanced log *P* integrates more consistently into the bilayer. This suggests that invasomes that utilize Limonene exhibit a higher degree of electrostatic repulsion, which results in a more stable dispersion.

### Effect on entrapment efficiency (EE%)

Entrapment efficiency was highest with cineole (83.57%, INV10), moderate with limonene (70.87%, INV7), and lowest with camphor (61.87%, INV11). Cineole's moderate log *P* allows for integration into both the aqueous interface and the lipid bilayer, resulting in stable drug encapsulation. Limonene's high log *P* causes excessive fluidization, which may initially improve drug loading but, at high concentrations, results in bilayer leakage and lower EE%. Camphor's lower log *P* and smaller molecular size may result in less efficient bilayer fluidization, limiting its ability to improve drug entrapment. Overall, the relationship between terpene concentration and EE% was nonlinear: low-to-moderate levels improved entrapment by increasing membrane fluidity, whereas high levels disrupted the bilayer and caused drug leakage, lowering EE%.

## Characterization of optimum Lido-loaded invasomes formulation

### Transmission electron microscopy (TEM)

**Fig. 4:**
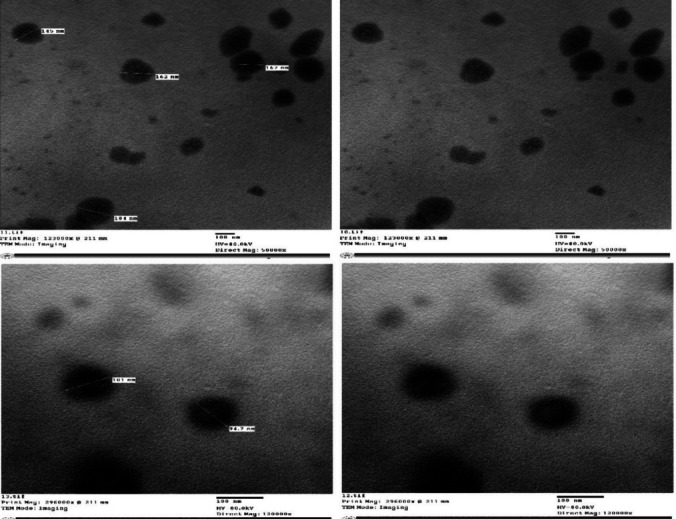
TEM photomicrograph of the optamized Lido-loaded invasomal formulation (INV10)

Using a transmission electron microscope (TEM), a morphological analysis of the optimized Lido-loaded invasomes was carried out. Invasomes that had been developed were unilamellar, with a uniform spherical shape that was discrete, and there was no fusion present, as shown in Fig. 4. As can be seen in the figure, the diameters of the vesicles that were seen in the micrographs are somewhat comparable to the values that were obtained through the process of particle size analysis.

### Fourier-transform infrared spectroscopy (FTIR)

**Fig. 5:**
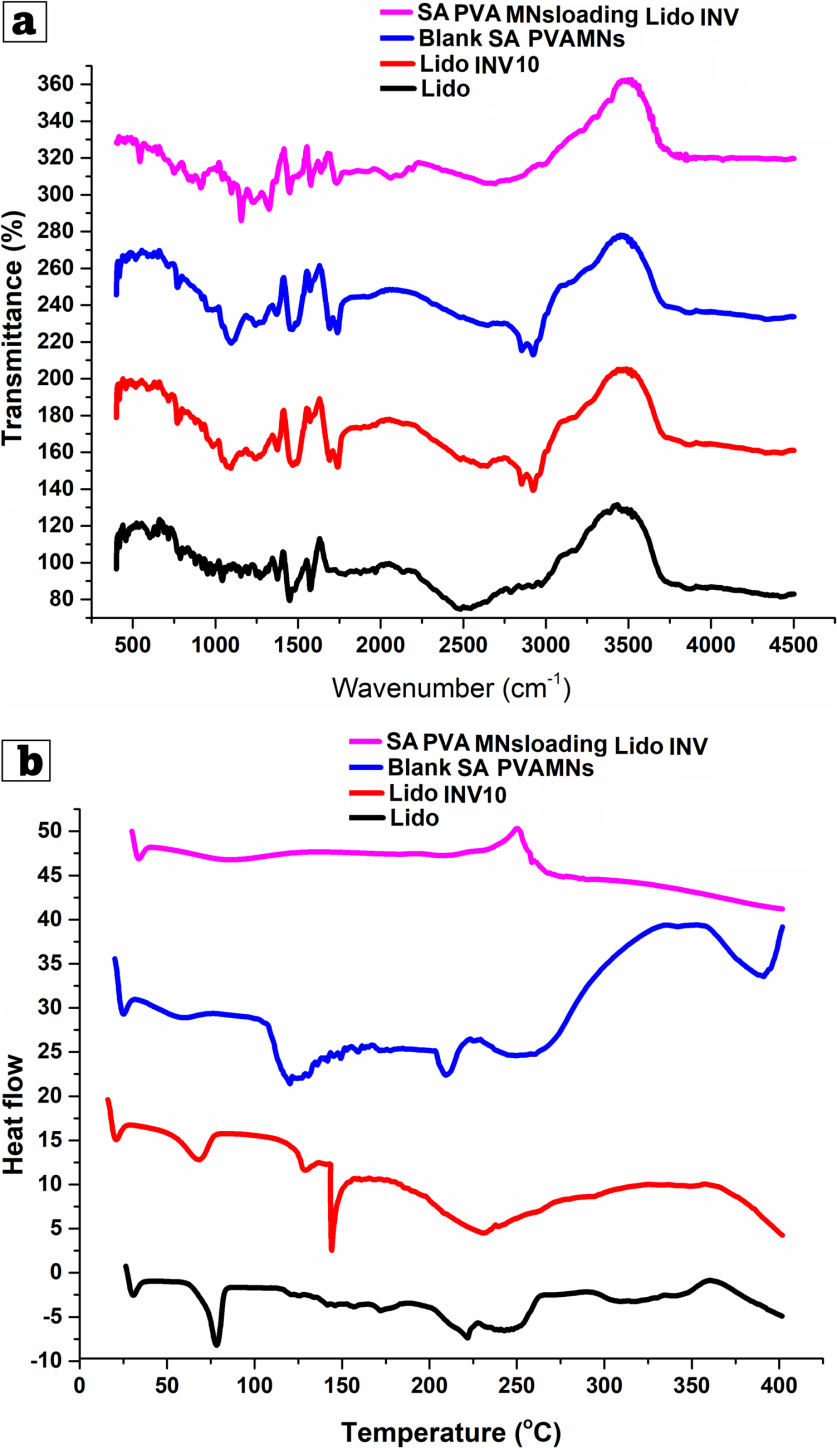
(**a**) Fourier-transform infrared spectroscopy (FTIR), and (**b**) Differential scanning calorimetry (DSC) for Lidocaine suspension (Lido), optimized Lido-loaded invasomes formulation (INV10), sodium alginate, polyvinyl alcohol (SA/PVA)-MNs loading freeze-dried Lido-loaded INV and blank MNs (sodium alginate, polyvinyl alcohol (SA/PVA)-MNs

Figure 5a shows the molecular interactions between Lido-loaded INV10, soft nanovesicles, and penetration enhancers by using FTIR. The drug was successfully incorporated and interacted with the lipid bilayer, as shown by the peak shifts and intensity variations in the spectra. Cineole formulations showed marked changes in the hydroxyl (-OH) stretching region (3200–3500 cm⁻^1^), indicating improved hydrogen bonding and membrane fluidization. Disrupted lipid packing makes Cineole-based vesicles smaller and more flexible. Invasomes made of Cineole exhibited peak shifts at 1700 cm⁻^1^ (C = O stretching) and 2850–2950 cm⁻^1^ (C-H stretching), indicating strong terpene-lipid bilayer interactions[53, 54]. This structural integration explains why bilayer expansion increases terpene concentrations and vesicle size. The presence of Lido in vesicles was confirmed by peaks at 1600–1650 cm⁻^1^, indicating aromatic C = C stretching, and around 1300 cm⁻^1^, indicating C-N A decrease in peak intensity in drug-loaded formulations indicates vesicle drug entrapment. Finally, the FTIR results show that Cineole increases membrane fluidity, Limonene and Camphor increase lipid bilayer interactions, which match particle size, stability, and entrapment efficiency[55].

### Differential scanning calorimetry (DSC)

Figure 5b shows the thermal properties of lidocaine suspension (Lido), and lidocaine-loaded invasomes (Lido-INV 10) using differential scanning calorimetry (DSC) thermograms. The high crystallinity of pure lidocaine (Lido) was demonstrated by the presence of a discernible endothermic peak at approximately 76.5 degrees Celsius, which corresponds to the melting point of the substance. Lidocaine-loaded invasomes (Lido-INV) exhibited a notable decrease in peak intensity, with the melting point either shifting slightly or vanishing, indicating that Lidocaine was effectively dispersed within the invasomal lipid bilayer, transitioning from a crystalline to a partially amorphous state[56].

## Short stability study for lidocaine invasomes

Table 4 shows A stability study assessed Lido-INV 10 stored at 4 °C and 25°Cover three months, concentrating on particle size (PS), polydispersity index (PDI), zeta potential (ZP), and entrapment efficiency (EE%). Particle size stability. The initial particle size of freshly prepared Lido-INV 10 was 295.8 ± 0.61 nm. After three months at 4 °C, the particle size (PS) increased to 299.5 ± 0.32 nm, whereas at 25 °C, it attained 303 ± 0.27 nm. An increase of less than 3% suggests a negligible aggregation effect over time, remaining within acceptable parameters. The newly formulated invasomes demonstrated a uniform size distribution, with a PDI of 0.267 ± 0.01. After storage, the PDI values rose to 0.291 ± 0.05 at 4 °C and 0.287 ± 0.01 at 25 °C, indicating slight changes in vesicle uniformity and stability. The initial ZP was −30.11 ± 0.98 mV, which remained stable at −30.41 ± 0.61 mV at 4 °C and −30.64 ± 0.91 mV at 25 °C. Invading particles preserved their surface charge, thus guaranteeing colloidal stability. The EE% of fresh Lido-INV 10 was 83.57 ± 0.43%, decreasing slightly to 83.43 ± 0.66% at 4 °C and significantly to 80 ± 0.12% at 25 °C. The observed slight reduction at elevated temperatures may be attributed to drug leakage from the lipid bilayer. Lido-INV 10 demonstrated vesicle integrity over three months, exhibiting minimal changes in PS, PDI, and ZP. A minor reduction in EE% at 25 °C indicates that 4 °C is more effective for drug encapsulation and minimizing leakage. Cineole enhances fluidity and preserves vesicle integrity, whereas phospholipids provide stabilization for Lido-INV 10. This study demonstrates that Lido-INV has a stability of three months, particularly at 4 °C, indicating its potential as a pharmaceutical formulation.

**Table 4:** The short-term stability results of optimized invasomes at 4 °C and 25 °C for 3 months. mean ± SD (n = 3)

Parameters	Lido-INV10 freshly prepared	Lido-INV10 after three months of storage at 4 °C	Lido-INV10 after three months of storage at 25 °C
PS (nm)	295.8 ± 0.61	299.5 ± 0.32	303 ± 0.27
PDI	0.267 ± 0.01	0.291 ± 0.05	0.287 ± 0.01
ZP (mV)	−30.11 ± 0.98	−30.41 ± 0.61	−30.64 ± 0.91
EE (%)	83.57 ± 0.43	83.43 ± 0.66	80 ± 0.12

Lido-INV: lidocaine invasomes

## Physical characteristics of the microneedles

### Mechanical strength and penetration capability test

**Fig. 6:**
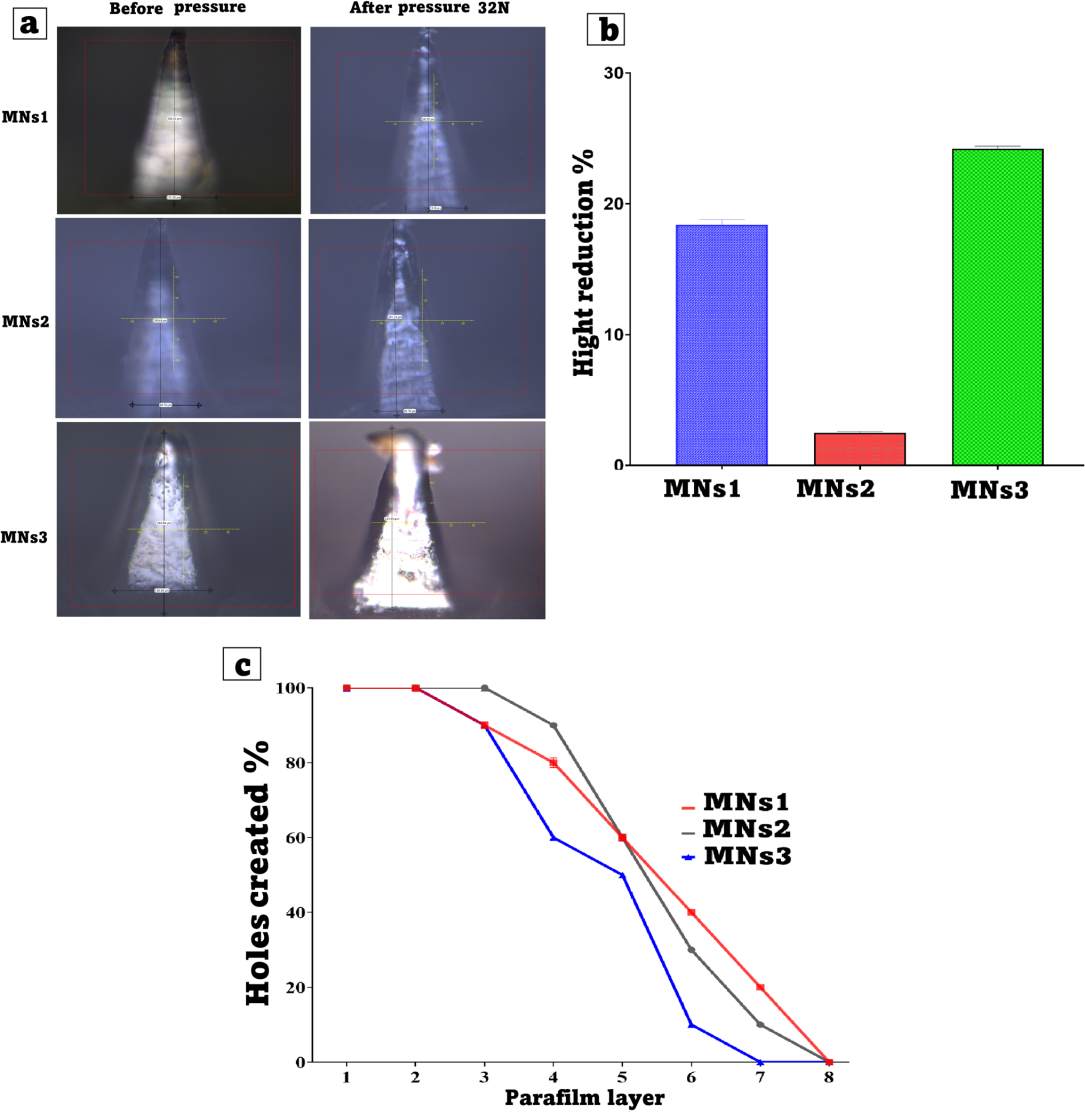
(**a**), (**b**)Mechanical Strength, and (**c**)Penetration capability for microneedles formulation (Mean ± SD, n = 3)

The purpose of the mechanical strength and penetration capability tests was to evaluate the resilience of MNs against compression and their ability to effectively penetrate Parafilm M^®^ as an artificial skin simulant that has been validated. Figure 6a and b illustrate the mechanical strength profile of the MNs formulation. The results show a relationship between Lido and polymer content and mechanical strength profile. MNs2 showed a significantly lower (p < 0.05) height reduction than MNs1 and MNs3. MNs1 demonstrated moderate height reduction, indicating balanced mechanical properties. The formulation contains a moderate amount of PVA (12%) and SA (20%), along with 40% Lido-INV, which is slightly higher than MNs2[56, 57]. The higher drug content may cause internal stress or heterogeneity in the matrix, resulting in slightly greater deformation under pressure (~ 15–18% height reduction). MNs2 had the lowest height reduction (~ 2–3%), indicating high mechanical strength. This is due to its high PVA (15%) and SA content (25%).SA carbohydrate polymer adds viscosity and structural integrity, while PVA is recognized for its ability to create strong hydrogen bonds, which enhances the film's rigidity. The 30% Lido-INV 10 load serves to enhance the matrix's integrity while ensuring that the drug content remains within manageable limits. MNs3 exhibited the most significant decrease in height, approximately 20–24%, suggesting a lack of mechanical integrity. Lido-INV, SA, and PVA exhibited the lowest percentages in this formulation, recorded at 50%, 15%, and 10%, respectively. Under compressive force, microneedles deform due to the inherent weakness of the crosslinked network and the inadequate structural support stemming from the low polymer content. The elevated drug load is expected to lead to further disturbances in polymer interactions[58].

The percentage of holes formed in Parafilm M^®^ is a direct indicator of the microneedles'(MNs) penetration efficiency and, indirectly, mechanical integrity (Fig. 6c). In this study, three formulations (MNs1, MNs2, and MNs3) were tested using an 8-layer Parafilm stack to simulate the skin's layered structure. The data revealed significant performance differences between the formulations, which can be attributed to their polymer-to-drug ratios and material composition. MNs2 had the highest penetration capability, with 100% perforation through the first three layers and 90% at the fourth layer. MNs2 reached 30% penetration even at layer six, suggesting that the formulation had sufficient mechanical strength to move force across more layers. With rather low standard deviation values, e.g., 0.3 to 0.9, indicating repeatable results, the consistency of performance across repetitions was also remarkable. MNs2's high polymer content, more especially, PVA (15%), sodium alginate (25%), which most certainly strengthened the microneedle matrix, helps to explain this improved performance. Moreover, the modest drug loading (30% Lido-INV) helped to preserve the structural integrity of the needles, to avoid tip deformation or breakage during use. In contrast, MNs1 demonstrated moderate penetration performance, with 100%-hole creation in the first two layers and a gradual decline to 80% at layer four and 40% at layer six. MNs1's standard deviation values were slightly higher than those of MNs2, particularly at layer four (SD = 1.3), indicating some performance variability. This formulation contained a balanced ratio of PVA (12%) and SA (20%), along with 40% Lido-INV. While this composition had adequate mechanical strength and flexibility, the higher drug content compared to MNs2 may have introduced microstructural heterogeneity, resulting in greater variability in penetration depth and mechanical resilience. MNs3 had the lowest penetration efficiency and the highest variability of the three formulations. Although initial performance at layers 1–2 was comparable to MNs1 and MNs2 (100% penetration), it quickly dropped to 60% at layer four and only 10% at layer six, with complete failure to penetrate beyond layer six. The low polymer content (PVA 10%), and the high drug load (50% Lido-INV) most certainly contribute to this poor performance. The high concentration of lyophilized drug might have undermined the polymer matrix, upset polymer crosslinking and reducing tip sharpness and strength. Higher standard deviations at some layers (SD = 0.6) at layer 6 indicate mechanical instability and uneven needle development. The results show that formulation composition affects microneedle performance. Too much drug loading weakens structures, but too much polymer strengthens and defines tips. MNs2 had the best balance of consistent penetration and mechanical performance, making it the most promising transmucosal delivery formulation

### Studies on drug content

Structural integrity and functional efficacy of microneedle formulations are much influenced by their drug content. Three microneedle formulations (MNs1, MNs2, and MNs3) were developed in the present work with different ratios of polymeric components, sodium alginate (SA), polyvinyl Alcohol (PVA), and 1% glycerin lidocaine-loaded invasomes. Using percentage composition by weight, the overall drug content was calculated to show MNs1 contained 70% Lido-INV, MNs2 contained 90%, and MNs3 contained the most at 91%. MNs2 showed the best mechanical performance and penetration efficiency while having the second-highest drug content. This formulation's high polymer content (25% sodium alginate and 15% PVA) most likely resulted in a cohesive matrix strong enough to support the microneedles under force. The structural integrity is maintained despite the substantial drug loading, a testament to the polymeric framework's ability to effectively mitigate the softening influence of the drug phase. Results show that MNs2 is the best candidate for further development because it combines mechanical reliability with increased drug loading. MNs1 exhibited a moderate level of mechanical performance when containing 70% drug content alongside reduced polymer concentrations. The reduced polymer content could have constrained the microneedle's capacity to maintain its shape when subjected to pressure, despite the lower drug content relative to MNs2, suggesting potentially enhanced structural properties. MNs1 thus displayed somewhat lower penetration and somewhat greater height reduction than MNs2. This implies that maintenance of performance depends on careful balancing of polymer composition and drug content. With just a somewhat higher drug content than MNs2 (91%), MNs3 showed the worst performance. With a low overall polymer proportion (15% SA and 10% PVA), it most certainly produced a weak, less cohesive matrix unable to preserve microneedle structure. The too high drug concentration with the available polymer could have upset molecular interactions and crosslinking, causing tip deformation and much lower penetration efficiency[23, 59]. MNs3 thus shows the negative consequence of too high a drug load without sufficient structural support. This study substantiates the notion that drug loading and matrix composition intricately influence microneedle efficacy. While therapeutic efficacy relies on elevated drug content, it must be complemented by sufficient polymer support to maintain mechanical integrity and functional performance. MNs2 is the optimal candidate for effective transmucosal drug delivery among the evaluated formulations, as it demonstrates a substantial drug load while maintaining mechanical integrity.

### Dissolution time test

**Fig. 7:**
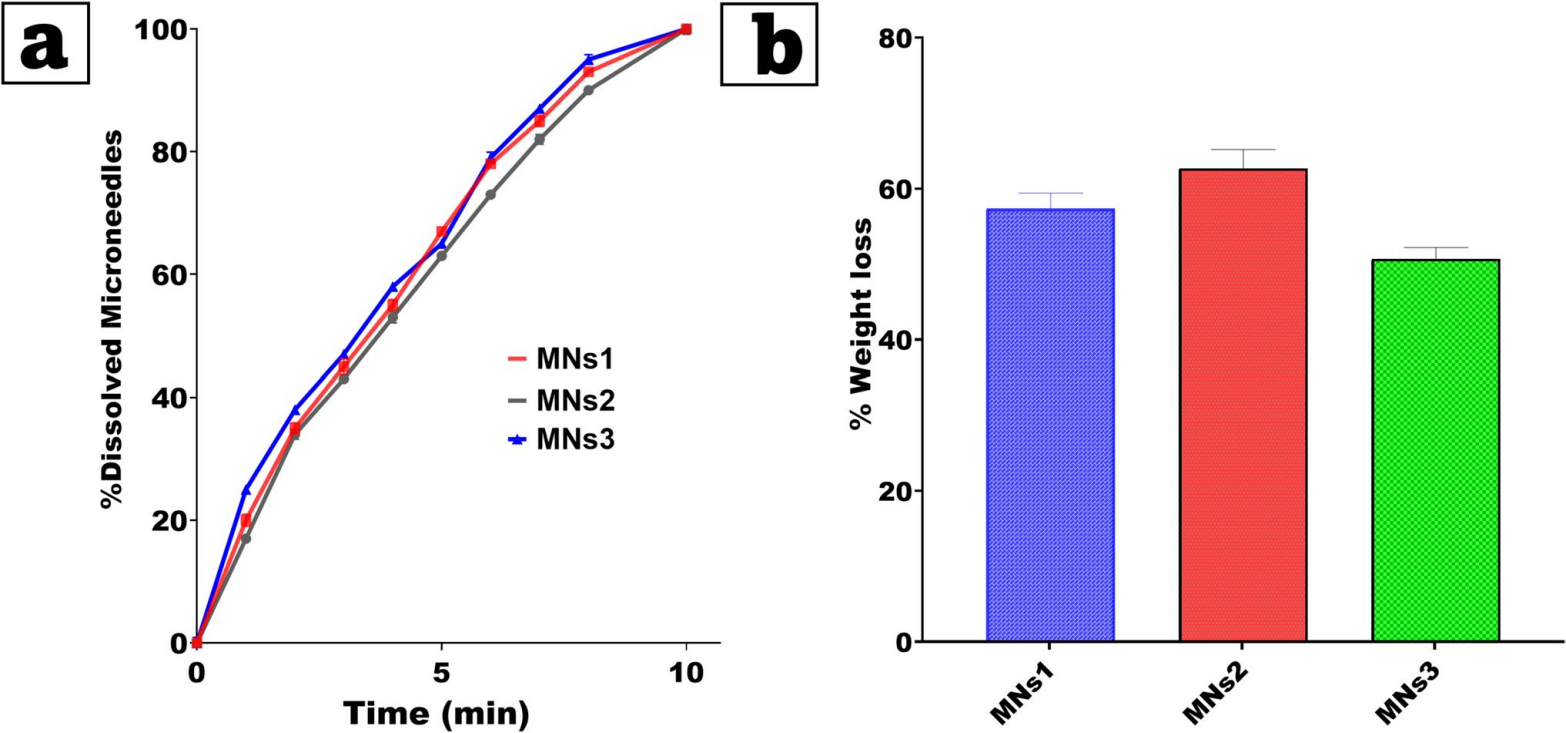
(**a**) Dissolution time, and (**b**) Water loss on drying (LOD) of MNs (Mean ± SD, n = 3)

Using the chicken pouch membrane model to mimic the buccal mucosal environment, as shown in Fig. 7a, we looked at how long it took for microneedles (MNs) to dissolve. All the tested formulations completely dissolved within 10 min of being used. The even dissolution seen across the whole thickness of the membrane shows that the MNs can get through and break down. Notably, there were no significant differences in the dissolution profile between polymer compositions or lidocaine concentrations. This consistency confirms that neither the polymer matrix ratio nor the active drug content has a significant influence on dissolution. Rather, the MN structure dissolves reliably when it meets the moist mucosal surface, allowing for localized drug delivery. These findings demonstrate that the rapid dissolution of MNs ensures timely drug release within the mucosa, as well as the delivery system's reproducibility and reliability across formulations. The homogeneity in dissolution profiles highlights the robustness of the MN design for effective mucosal application[60].

### Water loss on drying (LOD)

The loss on drying (LD) experiment was intended to determine the percentage of water that is lost during the drying stage of the microneedle (MN) manufacturing process. Figure 7b illustrates that all formulations were dried at room temperature for two days. No statistically significant differences (p > 0.05) were observed among the tested groups. This indicates that the drying process was consistent across formulations and successfully eliminated water. The recorded percentages of water loss, measured by weight change, are as follows: MNs1: 59%, 58%, 55%; MNs2: 60%, 65%, 63%; MNs3: 49%, 51%, 52%. MNs 1 (~ 57%), MNs 2 (~ 63%), had the highest average water loss; MNs3 had the lowest (~ 51%). These differences in polymer concentration and drug content could be the source, even though they are not statistically significant. MNs3 held more moisture, most probably from a less structured polymeric matrix and possible hygroscopicity with the lowest polymer content and highest drug load[61].

## Characterization of optimized microneedles

### Scanning electron microscopy (SEM)

**Fig. 8:**
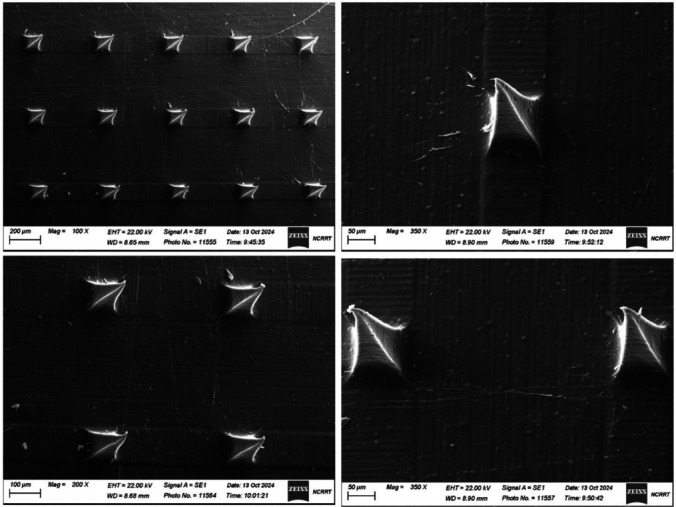
Scanning electron microscopy for optimized SA/PVA-MNs2

SEM was used to evaluate the surface morphology and microstructural integrity of optimized SA/PVA-MNs2 microneedles at different magnifications (100 ×, 200 ×, and 350 ×) as shown in Fig. 8. These images depict a well-organized, uniform microneedle array with sharp, pyramid-shaped needle tips, which are required for insertion into soft tissues such as the oral mucosa. At a 100-fold magnification, the array is free of structural defects, clustering, and disintegration. On delicate and uneven surfaces, like inflamed oral mucosa, uniformity is essential for proper depth of medication delivery. At 200 × and 350 × magnifications, microneedles can pass without harming well-defined structures, smooth surfaces, and pointed tips. A free SEM surface indicates that the polymer mixing, and Mold casting procedures were successful. Strong polymer crosslinks and mechanical strength of the dense, intact SA/PVA matrix allow microneedles to withstand application pressure[23]. Oral mucositis, a condition characterized by swelling and ulceration, requires the use of sharp, accurate needles for treatment. Because the area is so sensitive, the microneedles need to be very sharp so they can go through the mucosal layer without hurting it, and they also need to break down quickly so the therapeutic agent can get to where it needs to go. Sodium alginate facilitates the adherence of structures to living organisms. and PVA gives it strength and flexibility. Together, they create a structure that is both mechanically stable and biocompatible. Finally, SEM analysis reveals that the optimized SA/PVA-MNs2 formulation exhibits superior transmucosal delivery morphology. Its uniform geometry, sharp tip design, and structurally sound matrix make it a promising localized, painless drug administration method for oral mucositis therapy, with higher patient compliance.

### Fourier transform infrared (FTIR) analysis

Figure 5a show Thermal stability, chemical interactions, and successful incorporation of freeze-dried lidocaine-loaded invasomes into SA/PVA microneedles were studied using FTIR spectroscopy. The transmittance spectra of pure lidocaine, Lido INV10, blank SA/PVA-MNs, and those loaded with invasomes were compared from 400–4500 cm⁻^1^. Pure lidocaine FTIR spectrum reveals peaks at ~ 1650 cm⁻^1^ (amide C = O stretching), ~ 3300 cm⁻^1^ (N–H stretching), and ~ 2940 cm⁻^1^ (C-H stretching), confirming its chemical fingerprint The major lidocaine peaks in the Lido INV10 spectrum remain, indicating that the drug remains structurally intact after invasomes incorporation. Little variations in peak position and intensity during encapsulation, however, reveal weak interactions or hydrogen bonding among components of lipid vesicles. PVP, and alginate -OH stretching generates broad absorption bands between 3200 and 3400 cm⁻^1^ on the blue SA/PVA-MNs. They also have a clear peak around 1630 cm^−1^, which is caused by water or carboxylate group bending vibrations from sodium alginate[26, 62]. The 1100 cm⁻^1^ peak confirms the polymeric network structure, indicating C-O stretching vibrations from polysaccharide backbones. SA/PVA MNs containing lidocaine-invasomes exhibit polymer and lidocaine-specific peaks. Hydrogen bonding interactions or partial encapsulation of lidocaine in the microneedle matrix can be indicated by the shift or reduction in intensity of the -OH stretching region (~ 3300 cm⁻^1^) and the C = O region (~ 1650 cm). The spectral changes indicate that invasomes were integrated into the SA/PVA matrix. The retention of key lidocaine peaks suggests chemical stability during fabrication and freeze-drying. The FTIR results demonstrate that lidocaine-invasomes are thermally and structurally compatible with SA/PVA microneedles. Drug-loaded invasomes and the polymer matrix may improve drug stability and sustained release performance, both of which are essential for transmucosal oral ulcer treatment.

### Differential scanning calorimetry (DSC)

Figure 5b shows the thermal properties of freeze-dried lidocaine-invasomes incorporated into SA/PVA microneedles (SA/PVA-MNs), and blank SA/PVA-MNs were analyzed using differential scanning calorimetry (DSC) thermograms. The high crystallinity of lidocaine suspension (Lido) was demonstrated by the presence of a discernible endothermic peak at approximately 76.5 degrees Celsius, which corresponds to the melting point of the substance. Lidocaine-loaded invasomes (Lido-INV) exhibited a notable decrease in peak intensity, with the melting point either shifting slightly or vanishing, indicating that Lidocaine was effectively dispersed within the invasomal lipid bilayer, transitioning from a crystalline to a partially amorphous state. The freeze-dried Lido-INV 10 loaded in SA/PVA-MNs exhibited a broad endothermic peak at approximately 90–110 °C, indicating significant interactions between the invasomes and the polymeric matrix[11]. The crystalline of the drug was reduced even further because of this interaction, which also significantly improved the drug's stability. As a result of the absence of characteristic peaks in this region on the thermogram of the blank SA/PVA-MNs, it was determined that the control formulation did not contain any lidocaine. The thermal shifts and enthalpy reductions that were observed suggest that lido underwent a transition into an amorphous or molecularly dispersed state upon encapsulation. This resulted in an improvement in the drug's solubility, stability, and potential bioavailability when it was formulated as invasomes and incorporated into microneedles.

## Drug release study

### In vitro drug release study

**Fig. 9:**
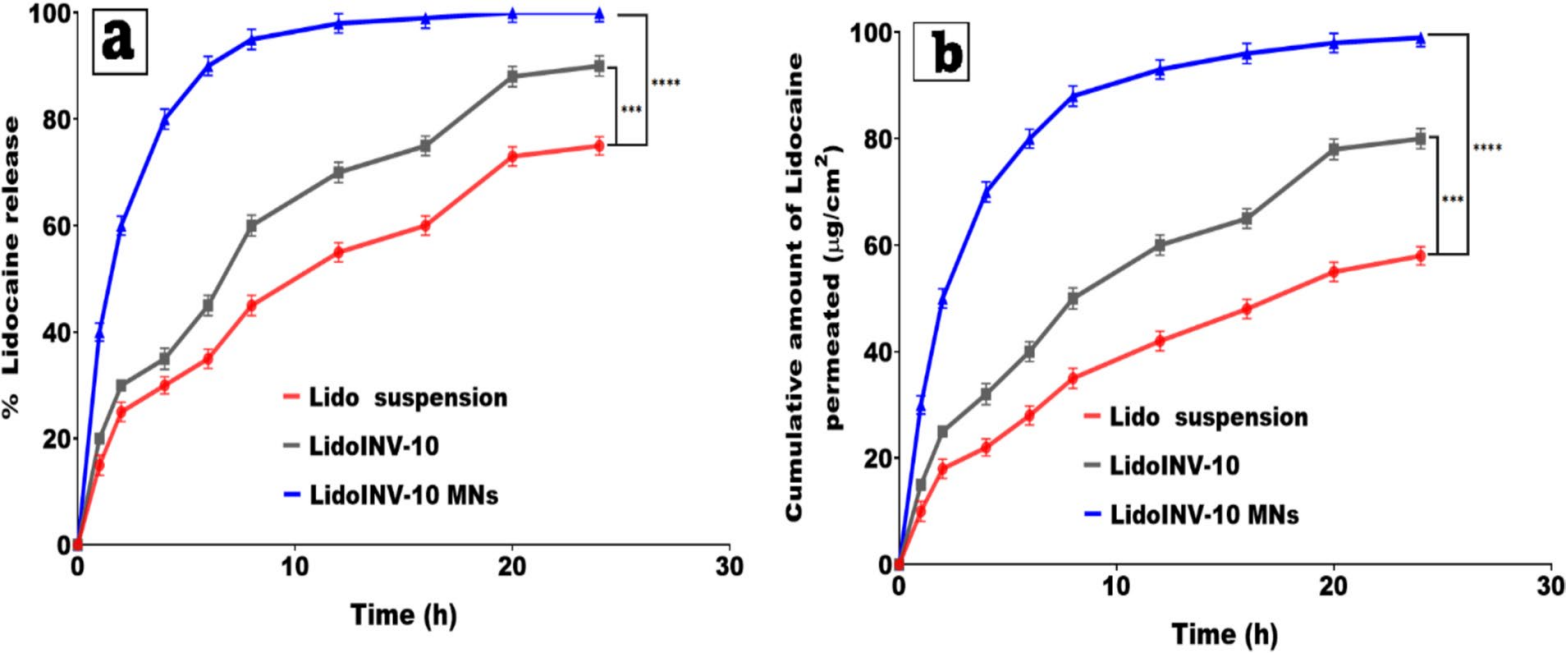
(**a**) In vitro drug release study, and (**b**) Ex-vivo permeation study for Lidocaine from Lidocaine suspension, Lidocaine invasomes (Lido INV-10), and NV-10 nanosuspension-loaded microneedles (Lido-INV-10 MNs)

The in vitro release profiles of lidocaine from different formulations, such as Lido suspension, LidoINV-10, and LidoINV-10 microneedle systems, were evaluated over a 24-h period as shown in Fig. 9a. The results indicate that the formulations demonstrate varying release behaviours, the Lido suspension exhibited a slow-release profile, achieving roughly 75% cumulative release within 24 h. The release profile of LidoINV-10 demonstrated a quicker and more consistent pattern, reaching around 90% within 24 h. In just 20 h, the LidoINV-10 MNs delivered almost the complete drug dose (100%), showcasing a remarkable drug release performance in comparison to both the suspension and the LidoINV-10. A moderate amount of Lido was released from the suspension in the first two to three hours; by the two-hour mark, about 25% had been released due to the rapid diffusion of the dissolved portion. Due to its low solubility, lidocaine was able to be released from the suspension at a slower rate, allowing for a more gradual release. LidoINV-10 demonstrated a consistent increase in release, likely attributable to the enhanced solubility and permeation characteristics of the invasomes system, which encapsulates the drug in a more bioavailable lipid vesicle format. The LidoINV-10 MNs demonstrated a significantly improved and expedited release profile, with 40% released in merely 1 h and 80% within 4 h. The enhanced performance is due to the microneedles'capacity to penetrate the barrier layer, facilitating direct drug delivery into the release medium and circumventing solubility constraints. The integration of the invasomes system within the microneedles may facilitate improved drug release by maintaining the drug in an amorphous or solubilized state, akin to the mechanism [39, 63]. The improved release characteristics of LidoINV-10, particularly the LidoINV-10 MNs, highlight the advantages of using microneedle technology and lipid-based carriers to increase the delivery and bioavailability of low-solubility drugs like lidocaine. Compared to the LidoINV-10 (60 ± 2.5%) and free lidocaine suspension (45 ± 2.0%), LidoINV-10 MNs had significantly higher Q24% values (95 ± 1.8%). At 18.8 ± 0.6% the steady-state flux (Jss) of LidoINV-10 MNs was noticeably higher than that of the free suspension (5.2 ± 0.3 μg/h·cm^2^). Improved drug transport across biological membranes has been achieved by means of the invasomes carrier system and the microneedle-mediated delivery mechanism, so generating better drug release and permeation profiles.

The in vitro release data were kinetically evaluated using four mathematical models: zero-order, first-order, Higuchi, and Korsmeyer-Peppas. Given the formulation design, which included the incorporation of lidocaine nanosuspension into dissolving microneedles made of hydrophilic polymers, diffusion and matrix dissolution were expected to play important roles[64]. The Higuchi model was particularly relevant since it describes drug release as the square root of a process that depends on Fickian diffusion from a homogeneous matrix and is time dependent. Using the Korsmeyer-Peppas model helped to improve understanding of the process since the differsive exponent (n value) indicates whether the release was entirely Fickian or a mix of diffusion and erosion-regulated processes[36]. Regarding microneedle-based systems, factors including polymer matrix dissolution and drug diffusion usually influence the non-linear release dynamics. Whereas first-order kinetics usually show concentration-dependent release and zero-order kinetics show constant release rates, based on the type of microneedle formulation, it was expected that the Higuchi or Korsmeyer-Peppas models would best fit the release data to help one better grasp the drug release behavior. The best values of the correlation coefficient (R^2^) of the suitable analysis guided the last choice[26].

### Ex-vivo permeation study

Figure 9b shows the ex vivo drug permeation profile of lidocaine from INV-10 nanosuspension-loaded microneedles (Lido-INV-10 MNs) compared to free lidocaine suspension[65]. The permeation study was carried out on fresh chicken pouch membranes, which served as a model for human buccal mucosa, using a modified Franz diffusion cell system. Compared to both the free suspension and the nanosuspension, the results demonstrated that Lido-INV-10 MNs considerably enhanced lidocaine permeation. The cumulative permeated amount of lidocaine in the microneedles group was approximately 99 µg/cm^2^ after 24 h, compared to 80 µg/cm^2^ in the Lido-INV-10 nanosuspension and only 58 µg/cm^2^ in the free lidocaine suspension. Approximately 30% of the medication permeated within the initial hour due to the microneedles, markedly surpassing the 10% and 15% permeation rates observed in the free suspension and nanosuspension groups, respectively. Relative to the other groups, Lido-INV-10 MNs exhibited a significantly elevated calculated apparent permeation coefficient (Kp), indicating that the microneedles effectively penetrate the mucosal barrier and facilitate drug transport. Along with mechanical creation of microchannels by the microneedles, the small particle size and enhanced solubility properties of the nanosuspension system may have helped to promote more direct and efficient transmembrane drug diffusion, so facilitating this enhancement[65]. The much higher total amount of lidocaine collected in the Lido-INV-10 MNs group after 24 h further demonstrated the creation of a drug reservoir within the mucosal tissue. With this localized reservoir, lidocaine can be kept at the target site for longer, which could mean less frequent reapplication and a longer duration of anaesthesia. This confirms what other studies have shown: that nano- and microneedle-based drug delivery systems are superior to traditional methods for increasing mucosal drug deposition and decreasing systemic exposure. Our formulations optimize localized drug action by combining microneedles and nanosuspension technology, making it especially useful for topical anaesthetic applications that require prolonged local efficacy with minimal systemic absorption. Thus, the Lido-INV-10 MNs formulation represents a promising approach to improving lidocaine delivery across mucosal tissues, with increased permeation, tissue deposition, and potentially superior therapeutic outcomes when compared to traditional delivery systems.

## In vivo study

### Biochemical assessment of inflammatory biomarkers

**Fig. 10:**
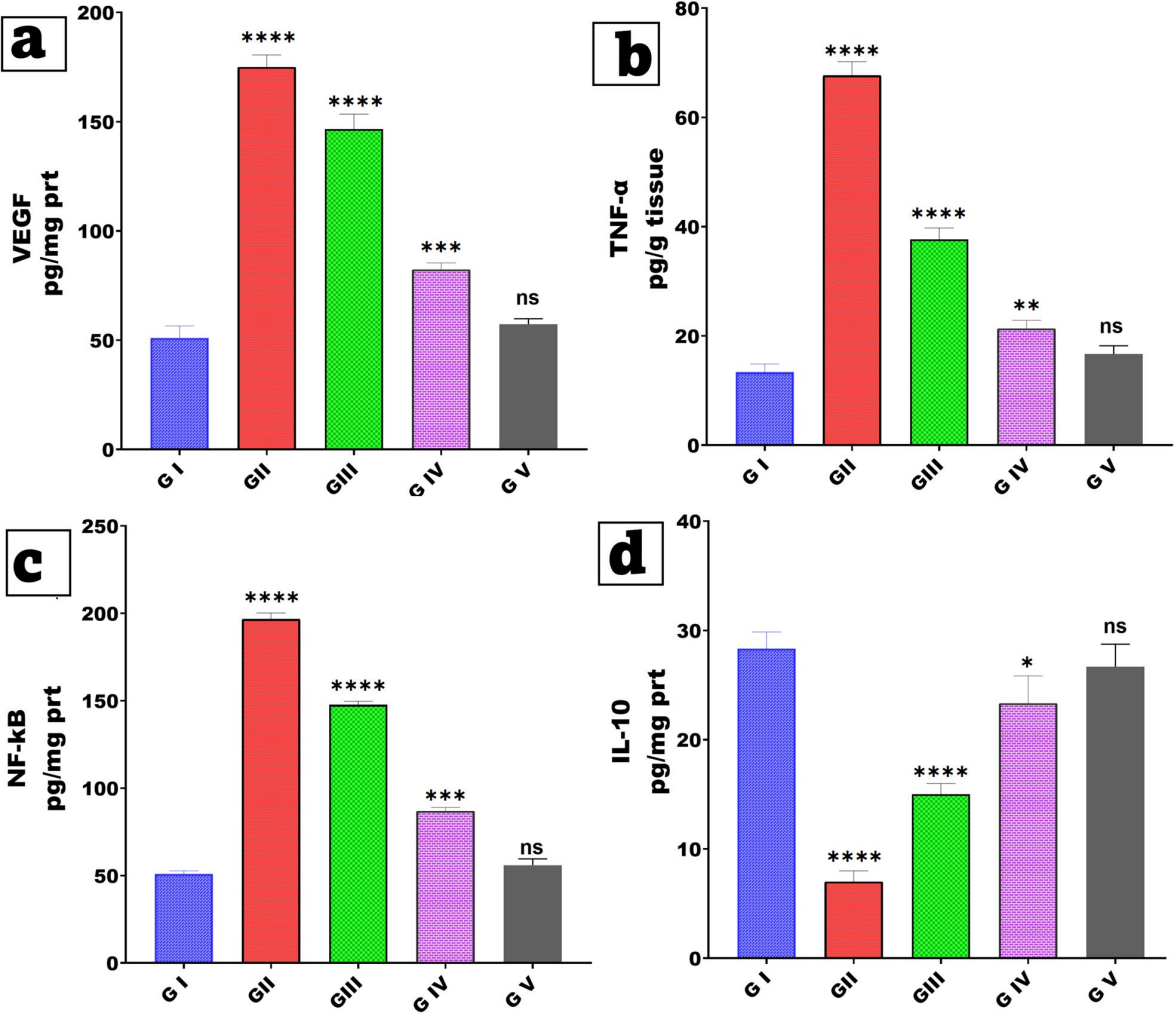
Inhibition of oral inflammation and promotion of Oral ulcerative mucositis healing in different treatment groups: (**a**–**d**) Serum levels of Vascular Endothelial Growth Factor (VEGF), Nuclear Factor kappa-light-chain-enhancer of activated B cells (NF-κB), Interleukin-10 (IL-10), and Tumor Necrosis Factor-alpha (TNF-α) as measured by ELISA. **Note**: Data are presented as Mean ± SD: *P < 0.05, **P < 0.01, ***P < 0.001, and ****P < 0.0001 versus the control group; ns (not significant) P > 0.05

Oral mucositis is a debilitating inflammatory condition of the mucosal lining that is commonly caused by chemotherapy, radiotherapy, or infections, resulting in pain, ulcers, and impaired healing. It is distinguished by a dysregulated inflammatory response, tissue damage, and excessive angiogenesis. Using ELISA assays, the study effectively created an oral mucosal model and evaluated systemic inflammatory and angiogenic markers (VEGF, TNF-α, NF-κB, and IL-10) to gauge disease severity and therapeutic responses as shown in Fig. 10. While IL-10 levels were noticeably lower (p < 0.0001), figure panels (a-d) show that Group II (disease treated control) had considerably higher levels of VEGF, TNF-α, and NF-κB than the healthy control Group I. This profile verified pathogenic angiogenesis (VEGF), NF-κB elevation, and severe mucosal inflammation including higher pro-inflammatory cytokine output. Once treatment was under way, the therapeutic groups differed significantly. Comparatively to the diseased control, Group III which received the standard formulation probably a nanosuspension, showed partial improvement in inflammatory markers, including notable but partial reductions in VEGF, TNF-α, and NF-κB levels, but IL-10 levels stayed much below those of healthy controls, suggesting a restricted anti-inflammatory response[66, 67]. The partial response could be ascribed to inadequate tissue penetration and a limited residence time of the free drug within the oral mucosa, so compromising the therapeutic efficacy. In contrast, Group IV, which received an advanced delivery system (most likely microneedles loaded with nanosuspension or optimized bioadhesive formulation), showed a significantly greater reduction in pro-inflammatory markers. VEGF, TNF-α, and NF-κB levels decreased significantly (p < 0.01–0.001), but IL-10 levels increased significantly (p < 0.05) compared to the diseased control[68]. This improved performance can be attributed to the advanced formulation's ability to increase drug penetration across the mucosal barrier, provide controlled and sustained drug release, and facilitate prolonged drug retention at the lesion site. The microchannel formation by microneedles, combined with the nanosuspension's increased solubility and bioavailability, most likely contributed to deeper tissue delivery and better modulation of inflammatory pathways. Group V, representing the optimized treatment group, achieved normalization of inflammatory and angiogenic markers, with no significant differences from the healthy control group. This complete restoration emphasizes the critical advantage of using localized delivery strategies capable of sustaining therapeutic levels over an extended period, minimizing systemic exposure, and efficiently modulating the mucosal inflammatory microenvironment[69, 70]. These findings indicate that the disease model induced significant systemic inflammation and that advanced formulation strategies, especially microneedle-based delivery or comparable enhanced systems, yielded superior therapeutic efficacy compared to traditional treatments. These systems offer significant potential for the effective management of oral mucositis and associated mucosal inflammatory disorders by facilitating enhanced mucosal penetration, establishing drug reservoirs within tissues, and providing sustained anti-inflammatory effects[30, 71].

### In vivo therapeutic efficiency of oral ulcerative mucositis and histopathological examination

**Fig. 11:**
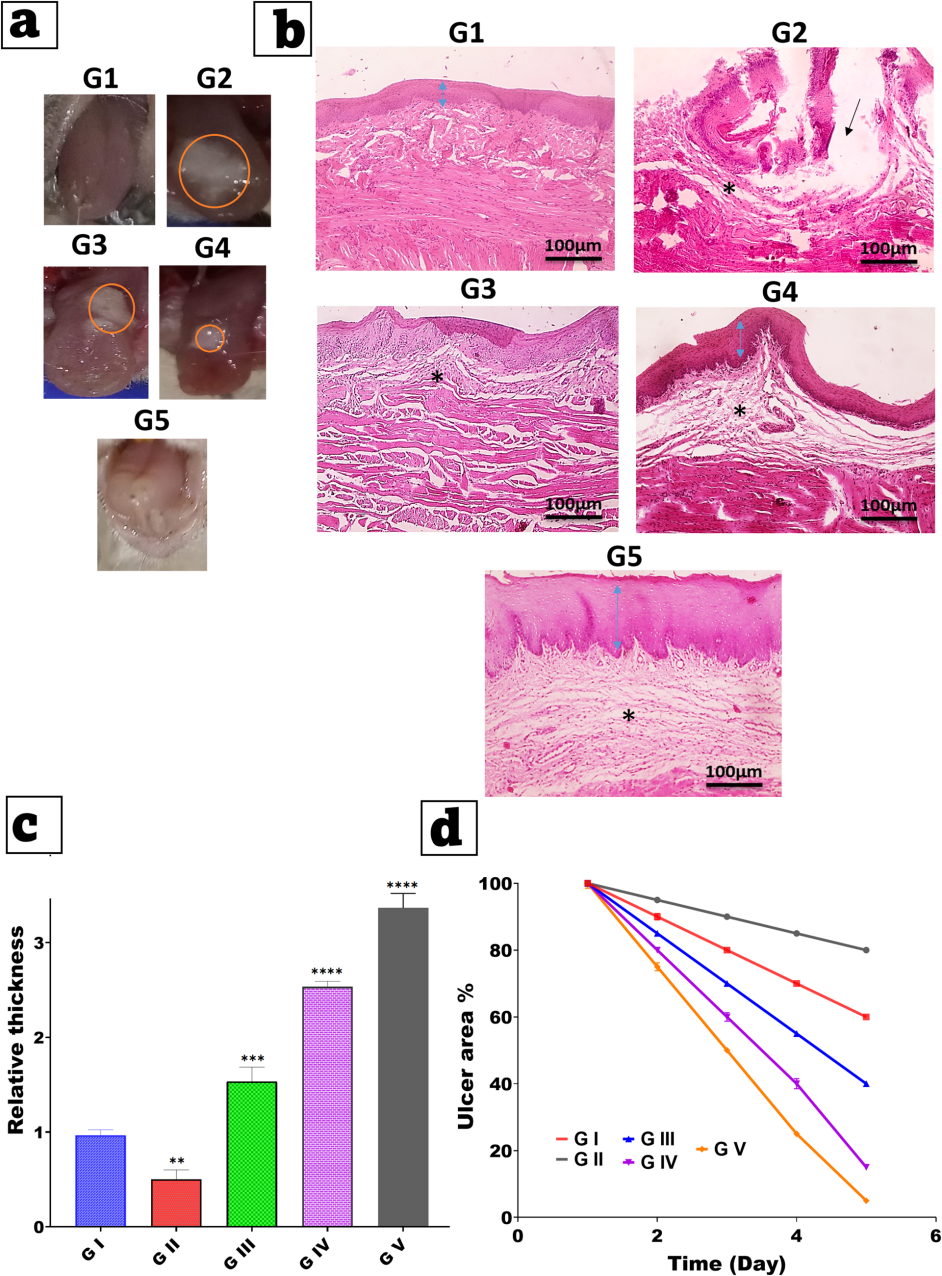
(**a**) Overall images of ulcers on day 5 following the intervention in different groups. (**b**) Hematoxylin and eosin-stained images of the ulcerated tissues H&E, X: 100 bar 100. (**c**) Comparative analysis of the ulcer area on day 5. (**d**) Quantitative analysis of mucosal epithelial layer thickness on day 5 (n = 5). Data are presented as Mean ± SD: **P < 0.01, ***P < 0.001, and ****P < 0.0001 versus the control group. **Note**: Group I (normal group, negative control), Group II (disease model group, positive control), and three treated groups: Group III (treated with a lidocaine solution), Group IV (treated with lidocaine loaded invasomes), and Group V (treated with an optimized microneedle patch)

Figure 11a shows the Images revealed significant differences between the five experimental groups. Group I (normal control) had intact oral mucosa with no ulceration, whereas Group II (disease model) had a prominent ulcer with visible inflammation and tissue degradation. In contrast, Group III (lidocaine solution) and IV (lidocaine-loaded invasomes) showed some improvement in ulcer size and appearance. Most notably, Group V, which received the optimized microneedle (MN) patch, demonstrated nearly complete ulcer healing and visibly restored mucosal integrity. These visual observations suggest that the microneedle-treated group had higher healing efficiency[72, 73]**.**

Figure 11b show the microscopic pictures of tongue sections showing normal histology of keratinized stratified squamous epithelial tissue, submucosa and muscular layer in control normal rabbits. Ulceration in covering mucosa (thin black arrow) with deposition of submucosal granulation tissue (*) are seen in control + ve. Three days after wound induction, regenerated healed epithelium with deposition of excess granulation tissue (*) are seen in treated group with Lido-INV& Lido-INV 10 loaded in SA/PVA-MNs, intact mucosa with deposition of less submucosal granulation (*) are seen in treated group with micro. Group I had normal histological architecture, including well-organized stratified squamous epithelium and dense connective tissue. Group II, on the other hand, demonstrated severe epithelial disruption, ulceration, and significant inflammatory cell infiltration, indicating that ulcer induction was successful. Group III showed partial epithelial regeneration, but there were still signs of inflammation. Group IV showed improved epithelial continuity and reduced inflammation, indicating better healing. Group V showed the most significant histological restoration, with a nearly complete epithelial layer, minimal inflammatory infiltration, and well-aligned connective tissues[74, 75]. These histological features provide strong support for the MN patch's superior efficacy in promoting mucosal repair.

Figure 11c shows the quantitatively compares epithelial thickness between groups, indicating the degree of mucosal regeneration. The disease group (GII) had the smallest relative thickness, whereas treatment groups (GIII-GV) showed progressively greater epithelial restoration. Group V had the highest epithelial thickness with a statistically significant increase compared to all other groups (***P < 0.0001), confirming previous findings that Lido-INV 10 loaded in SA/PVA-MNs enhances epithelial regeneration. This parameter provides additional evidence that microneedle-based drug delivery promotes rapid and efficient tissue recovery[60].

Finally, Fig. 11d depicts a time-dependent reduction in ulcer areas across all groups. Group II had the slowest healing rate, with very little reduction in ulcer size by day 5. Groups III and IV showed moderate improvements, whereas Group V had the fastest and most significant decrease in ulcer area, approaching near-complete resolution. This finding implies that the optimized microneedle patch not only accelerates epithelial healing, but also significantly reduces the overall ulcer burden in a shorter period [61].

## Conclusion

This work successfully developed and optimized a novel lidocaine-loaded invasomal system incorporated into dissolving sodium alginate/polyvinyl alcohol (SA/PVA) microneedles for better treatment of oral ulcerative mucositis. The optimal invasomes with cineole terpene had a small particle size (~ 295 nm), narrow polydispersity index (~ 0.267), good colloidal stability (ZP ~ −30 mV), high entrapment efficiency (83.57%), and great stability over three months at 4 °C. Microneedle patches produced with an optimal formulation (MNs2) exhibited superior mechanical strength, excellent mucosal penetration, rapid dissolution profiles in oral tissues, and high drug loading efficiency. In both in -vitro and ex-vivo studies, Lido-INV-10 MNs demonstrated superior drug release (~ 95% within 24 h) and permeation (~ 99 µg/cm^2^ across buccal mucosa) compared to conventional lidocaine suspension and nanosuspension formulations. Kinetic modeling revealed that drug release followed the Higuchi and Korsmeyer-Peppas models, implying diffusion and erosion control mechanisms. In an in-vivo oral mucositis model, the microneedle system accelerated mucosal healing, normalized inflammatory and angiogenic markers (VEGF, TNF-α, NF-κB, and IL-10), and achieved nearly complete ulcer recovery when compared to standard treatments. Histopathological analysis revealed that microneedle treatment significantly restored tissue function. Invasomes nano-vesicles and dissolving microneedles form a potent localized drug delivery system for oral mucositis. This platform improved lidocaine bioavailability sustained local drug release, modulation of inflammatory pathways, and mucosal tissue regeneration. The medical application of the novel, minimally invasive, and patient-friendly microneedle patches for oral mucosal diseases is promising**.**

## Data Availability

The authors confirm that the data supporting the findings of this study are available within the article.
